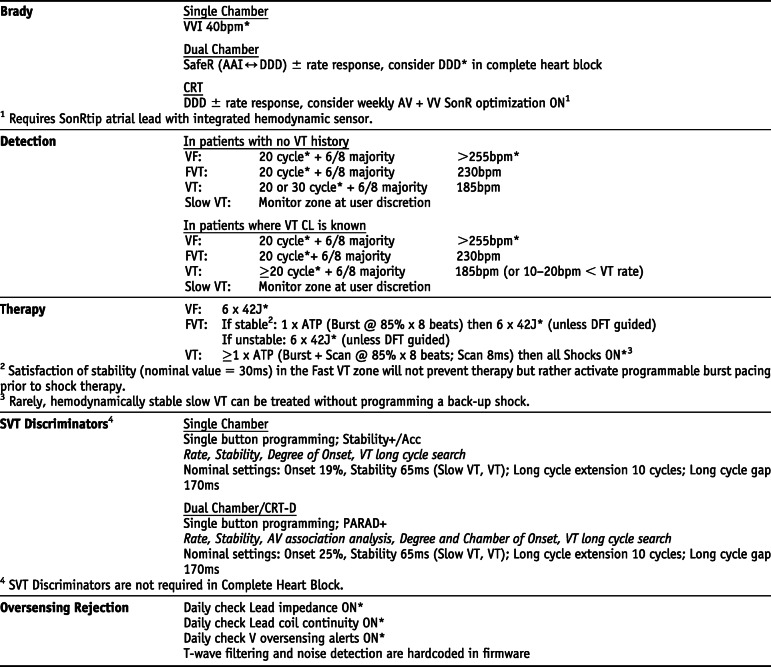# 2019 HRS/EHRA/APHRS/LAHRS focused update to 2015 expert consensus statement on optimal implantable cardioverter-defibrillator programming and testing

**DOI:** 10.1007/s10840-019-00662-4

**Published:** 2020-01-21

**Authors:** Martin K. Stiles, Laurent Fauchier, Carlos A. Morillo, Bruce L. Wilkoff

**Affiliations:** 1 Waikato Hospital, Hamilton, New Zealand; 2 Centre Hospitalier Universitaire Trousseau, Université François Rabelais, Tours, France; 3 Libin Cardiovascular Institute of Alberta, University of Calgary, Calgary, Canada; 4 Cleveland Clinic, Cleveland, OH USA

**Keywords:** Antitachycardia pacing, Bradycardia mode and rate, Defibrillation testing, Implantable cardioverter-defibrillator, Programming, Sudden cardiac death, Tachycardia detection, Tachycardia therapy, Ventricular tachycardia, Ventricular fibrillation

## Abstract

The *2015 HRS/EHRA/APHRS/SOLAECE Expert Consensus Statement on Optimal Implantable Cardioverter-Defibrillator Programming and Testing* provided guidance on bradycardia programming, tachycardia detection, tachycardia therapy, and defibrillation testing for implantable cardioverter-defibrillator (ICD) patient treatment. The 32 recommendations represented the consensus opinion of the writing group, graded by Class of Recommendation and Level of Evidence. In addition, Appendix B provided manufacturer-specific translations of these recommendations into clinical practice consistent with the recommendations within the parent document. In some instances, programming guided by quality evidence gained from studies performed in devices from some manufacturers was translated such that this programming was approximated in another manufacturer’s ICD programming settings. The authors found that the data, although not formally tested, were strong, consistent, and generalizable beyond the specific manufacturer and model of ICD. As expected, because these recommendations represented strategic choices to balance risks, there have been reports in which adverse outcomes were documented with ICDs programmed to Appendix B recommendations. The recommendations have been reviewed and updated to minimize such adverse events. Notably, patients who do not receive unnecessary ICD therapy are not aware of being spared potential harm, whereas patients in whom their ICD failed to treat life-threatening arrhythmias have their event recorded in detail. The revised recommendations employ the principle that the randomized trials and large registry data should guide programming more than anecdotal evidence. These recommendations should not replace the opinion of the treating physician who has considered the patient’s clinical status and desired outcome via a shared clinical decision-making process.

**Document Reviewers:** Serge Boveda, MD, PhD; Michael R. Gold, MD, PhD, FHRS; Roberto Keegan, MD; Valentina Kutyifa, MD, PhD, FHRS, FESC, FACC; Chu-Pak Lau, MD, FHRS, CCDS; Mark A. McGuire, MBBS, PhD; Siva K. Mulpuru, MD, FHRS, CCDS; David J. Slotwiner, MD, FHRS; William Uribe, MD, MBA, FHRS.

**TABLE OF CONTENTS**

Abstract………………………………………*In this issue*

Manufacturer-Specific Translation of ICD Programming Recommendations: Abbott (Formerly St. Jude Medical)………………………………………....*In this issue*

Manufacturer-Specific Translation of ICD Programming Recommendations: BIOTRONIK……………….*In this issue*

Manufacturer-Specific Translation of ICD Programming Recommendations: Boston Scientific……………*In this issue*

Manufacturer-Specific Translation of ICD Programming Recommendations: Medtronic…………………..*In this issue*

Manufacturer-Specific Translation of ICD Programming Recommendations: MicroPort CRM (Formerly LivaNova and Sorin Group)………………………………...*In this issue*

Appendix 1 Author disclosure table………….*In this issue*

Appendix 2 Reviewer disclosure table………..*In this issue*

## Manufacturer-specific translation of ICD programming recommendations^‡^

^‡^The manufacturer-specific programming settings/choices set forth below are based on a compilation of clinical expertise and clinical trial data as reported in the *2015 HRS/EHRA/APHRS/SOLAECE Expert Consensus Statement on Optimal Implantable Cardioverter-Defibrillator Programming and Testing*, of which this Appendix B is a part. These recommended settings/choices represent a diligent and good faith effort on the part of the writing committee to translate the consensus statement recommendations to device settings/choices for the four specified clinical issues/implantable cardioverter-defibrillator (ICD) therapies where the writing committee considered that there was sufficient consensus and supporting data to make recommendations intended to improve the safety, morbidity, and mortality profile of patients with these clinical issues/ICD therapies. They are the recommendations of the writing committee only. They do not represent the position or recommendations of HRS, EHRA, LAHRS (formerly SOLAECE), or APHRS, nor are they the manufacturer’s nominal settings or the precise programming tested during clinical trials of these devices, nor are they necessarily the settings/choices that would be recommended by the manufacturer. These recommended settings/choices are not applicable in all circumstances. As stated in the Introduction to the consensus statement, “The care of individual patients must be provided in context of their specific clinical condition and the data available on that patient.” Each treating physician must carefully consider the circumstances of their individual patient and determine whether these recommended settings/choices are appropriate to their patient’s circumstances.

### Abbott (Formerly St. Jude Medical) *Settings that are not nominal are marked with an asterisk

**Figure:**
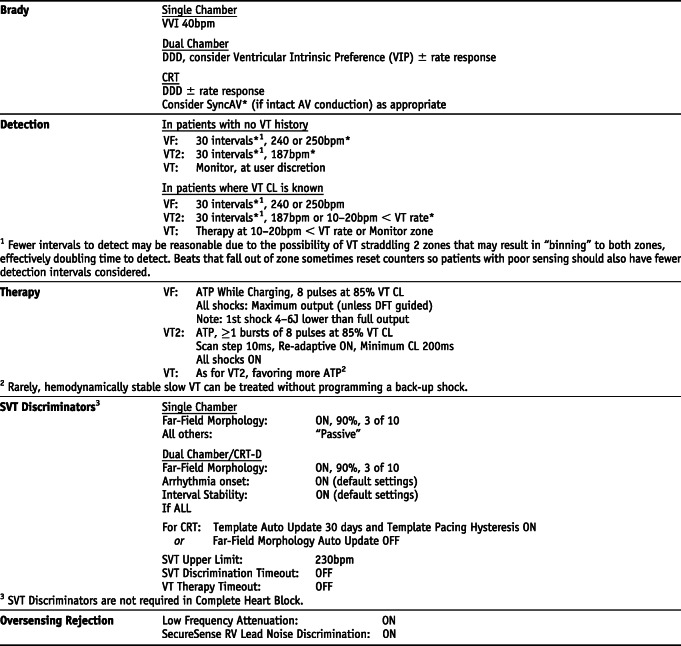

## Manufacturer-specific translation of ICD programming recommendations^‡^

^‡^The manufacturer-specific programming settings/choices set forth below are based on a compilation of clinical expertise and clinical trial data as reported in the *2015 HRS/EHRA/APHRS/SOLAECE Expert Consensus Statement on Optimal Implantable Cardioverter-Defibrillator Programming and Testing*, of which this Appendix B is a part. These recommended settings/choices represent a diligent and good faith effort on the part of the writing committee to translate the consensus statement recommendations to device settings/choices for the four specified clinical issues/implantable cardioverter-defibrillator (ICD) therapies where the writing committee considered that there was sufficient consensus and supporting data to make recommendations intended to improve the safety, morbidity, and mortality profile of patients with these clinical issues/ICD therapies. They are the recommendations of the writing committee only. They do not represent the position or recommendations of HRS, EHRA, LAHRS (formerly SOLAECE), or APHRS, nor are they the manufacturer’s nominal settings or the precise programming tested during clinical trials of these devices, nor are they necessarily the settings/choices that would be recommended by the manufacturer. These recommended settings/choices are not applicable in all circumstances. As stated in the Introduction to the consensus statement, “The care of individual patients must be provided in context of their specific clinical condition and the data available on that patient.” Each treating physician must carefully consider the circumstances of their individual patient and determine whether these recommended settings/choices are appropriate to their patient’s circumstances.

### BIOTRONIK *Settings that are not nominal are marked with an asterisk

**Figure:**
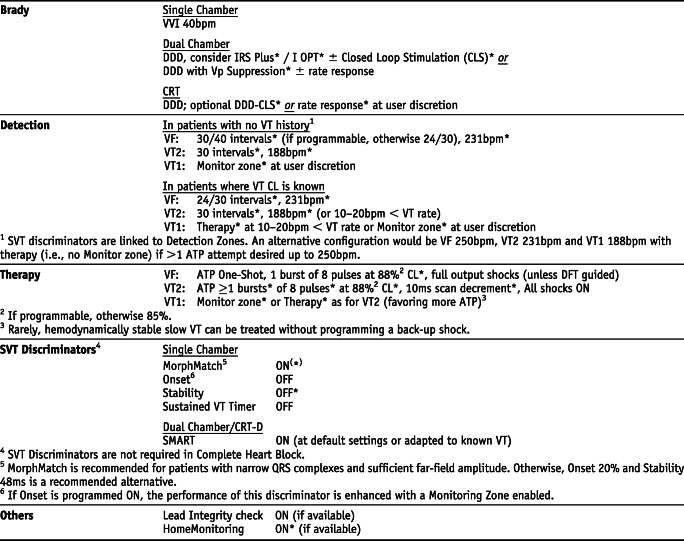

## Manufacturer-specific translation of ICD programming recommendations^‡^

^‡^The manufacturer-specific programming settings/choices set forth below are based on a compilation of clinical expertise and clinical trial data as reported in the *2015 HRS/EHRA/APHRS/SOLAECE Expert Consensus Statement on Optimal Implantable Cardioverter-Defibrillator Programming and Testing*, of which this Appendix B is a part. These recommended settings/choices represent a diligent and good faith effort on the part of the writing committee to translate the consensus statement recommendations to device settings/choices for the four specified clinical issues/implantable cardioverter-defibrillator (ICD) therapies where the writing committee considered that there was sufficient consensus and supporting data to make recommendations intended to improve the safety, morbidity, and mortality profile of patients with these clinical issues/ICD therapies. They are the recommendations of the writing committee only. They do not represent the position or recommendations of HRS, EHRA, LAHRS (formerly SOLAECE), or APHRS, nor are they the manufacturer’s nominal settings or the precise programming tested during clinical trials of these devices, nor are they necessarily the settings/choices that would be recommended by the manufacturer. These recommended settings/choices are not applicable in all circumstances. As stated in the Introduction to the consensus statement, “The care of individual patients must be provided in context of their specific clinical condition and the data available on that patient.” Each treating physician must carefully consider the circumstances of their individual patient and determine whether these recommended settings/choices are appropriate to their patient’s circumstances.

### Boston Scientific *Settings that are not nominal are marked with an asterisk

**Figure:**
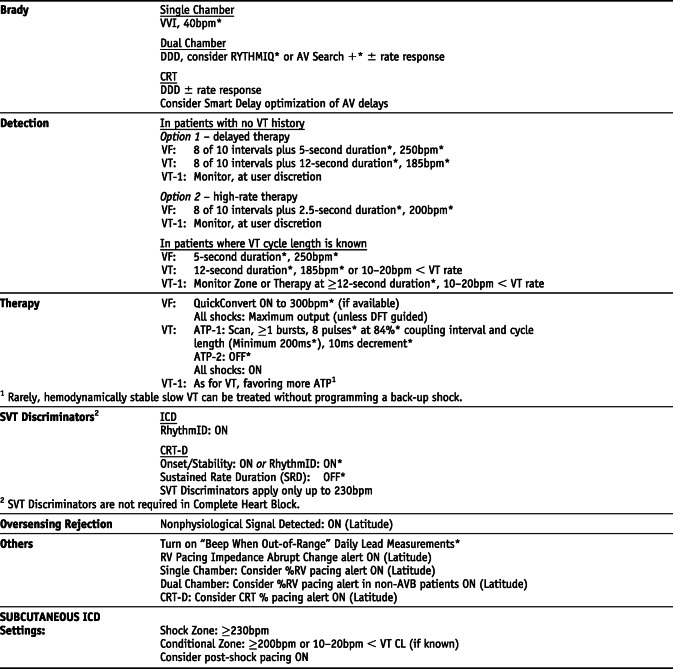

## Manufacturer-specific translation of ICD programming recommendations^‡^

^‡^The manufacturer-specific programming settings/choices set forth below are based on a compilation of clinical expertise and clinical trial data as reported in the *2015 HRS/EHRA/APHRS/SOLAECE Expert Consensus Statement on Optimal Implantable Cardioverter-Defibrillator Programming and Testing*, of which this Appendix B is a part. These recommended settings/choices represent a diligent and good faith effort on the part of the writing committee to translate the consensus statement recommendations to device settings/choices for the four specified clinical issues/implantable cardioverter-defibrillator (ICD) therapies where the writing committee considered that there was sufficient consensus and supporting data to make recommendations intended to improve the safety, morbidity, and mortality profile of patients with these clinical issues/ICD therapies. They are the recommendations of the writing committee only. They do not represent the position or recommendations of HRS, EHRA, LAHRS (formerly SOLAECE), or APHRS, nor are they the manufacturer’s nominal settings or the precise programming tested during clinical trials of these devices, nor are they necessarily the settings/choices that would be recommended by the manufacturer. These recommended settings/choices are not applicable in all circumstances. As stated in the Introduction to the consensus statement, “The care of individual patients must be provided in context of their specific clinical condition and the data available on that patient.” Each treating physician must carefully consider the circumstances of their individual patient and determine whether these recommended settings/choices are appropriate to their patient’s circumstances.

### Medtronic *Settings that are not nominal are marked with an asterisk

**Figure:**
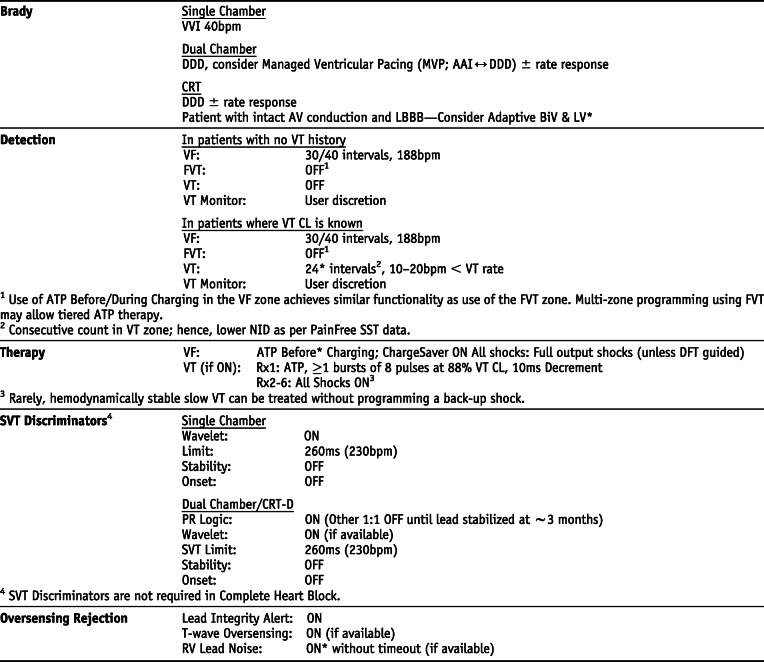

## Manufacturer-specific translation of ICD programming recommendations^‡^

^‡^The manufacturer-specific programming settings/choices set forth below are based on a compilation of clinical expertise and clinical trial data as reported in the *2015 HRS/EHRA/APHRS/SOLAECE Expert Consensus Statement on Optimal Implantable Cardioverter-Defibrillator Programming and Testing*, of which this Appendix B is a part. These recommended settings/choices represent a diligent and good faith effort on the part of the writing committee to translate the consensus statement recommendations to device settings/choices for the four specified clinical issues/implantable cardioverter-defibrillator (ICD) therapies where the writing committee considered that there was sufficient consensus and supporting data to make recommendations intended to improve the safety, morbidity, and mortality profile of patients with these clinical issues/ICD therapies. They are the recommendations of the writing committee only. They do not represent the position or recommendations of HRS, EHRA, LAHRS (formerly SOLAECE), or APHRS, nor are they the manufacturer’s nominal settings or the precise programming tested during clinical trials of these devices, nor are they necessarily the settings/choices that would be recommended by the manufacturer. These recommended settings/choices are not applicable in all circumstances. As stated in the Introduction to the consensus statement, “The care of individual patients must be provided in context of their specific clinical condition and the data available on that patient.” Each treating physician must carefully consider the circumstances of their individual patient and determine whether these recommended settings/choices are appropriate to their patient’s circumstances.

### MicroPort CRM (Formerly LivaNova and Sorin Group) *Settings that are not nominal are marked with an asterisk

**Figure:**